# Plant-derived nanomaterials (PDNM): a review on pharmacological potentials against pathogenic microbes, antimicrobial resistance (AMR) and some metabolic diseases

**DOI:** 10.1007/s13205-023-03713-w

**Published:** 2023-08-04

**Authors:** Emmanuel Sunday Okeke, Ekene John Nweze, Emeka Godwin Anaduaka, Charles Obinwanne Okoye, Chioma Assumpta Anosike, Parker Elijah Joshua, Timothy Prince Chidike Ezeorba

**Affiliations:** 1 Department of Biochemistry, Faculty of Biological Sciences, University of Nigeria, Nsukka, 410001 Enugu Nigeria; 2 Natural Science Unit, School of General Studies, University of Nigeria, Nsukka, 410001 Enugu Nigeria; 3 Institute of Environmental Health and Ecological Security, School of Environment and Safety Engineering, Jiangsu University, Zhenjiang, 212013 Jiangsu China; 4 School of Environment and Safety Engineering, Jiangsu University, Zhenjiang, 212013 People’s Republic of China; 5 Department of Zoology and Environmental Biology, University of Nigeria, Nsukka, 410001 Enugu Nigeria; 6 Biofuels Institute, Jiangsu University, Zhenjiang, 212013 People’s Republic of China; 7 Department of Genetics and Biotechnology, Faculty of Biological Sciences, University of Nigeria, Nsukka, 410001 Enugu Nigeria; 8 Department of Environmental Health and Risk Management, College of Life and Environmental Sciences, University of Birmingham Edgbaston, Birmingham, B15 2TT UK

**Keywords:** Plant-derived nanomaterials (PDNMs), Metabolic diseases, Green nanoparticles, Antimicrobial resistance (AMR), Drug delivery

## Abstract

Plant-derived nanomaterials (PDNM) have gained significant attention recently due to their potential pharmacological applications against pathogenic microbes, antimicrobial resistance (AMR), and certain metabolic diseases. This review introduces the concept of PDNMs and their unique properties, including their small size, high surface area, and ability to penetrate biological barriers. Besides various methods for synthesizing PDNMs, such as green synthesis techniques that utilize plant extracts and natural compounds, the advantages of using plant-derived materials, such as their biocompatibility, biodegradability, and low toxicity, were elucidated. In addition, it examines the recent and emerging trends in nanomaterials derived from plant approaches to combat antimicrobial resistance and metabolic diseases. The sizes of nanomaterials and their surface areas are vital as they play essential roles in the interactions and relationships between these materials and the biological components or organization. We critically analyze the biomedical applications of nanoparticles which include antibacterial composites for implantable devices and nanosystems to combat antimicrobial resistance, enhance antibiotic delivery, and improve microbial diagnostic/detection systemsIn addition, plant extracts can potentially interfere with metabolic syndrome pathways; hence most nano-formulations can reduce chronic inflammation, insulin resistance, oxidative stress, lipid profile, and antimicrobial resistance. As a result, these innovative plant-based nanosystems may be a promising contender for various pharmacological applications.

## Introduction

Nanoparticles (NPs) have become a prominent area of research in recent years due to their extensive uses in various fields, including diagnostics, biomarkers, cell identification, antimicrobials, drug administration, and cancer therapy (Adeniji et al. 2022). Nanoparticles can be synthesized and developed via physical, chemical, and biological processes. On the other hand, biological approaches are particularly appealing since they are simple, inexpensive, and can be adjusted to produce the required shape, size, and functionality (Singh et al. 2018; Trivedi et al. 2022a).

In the top-down method, physical processes such as grinding, diffusion, thermal decomposition, irradiation, and others break the bulky material into small particles. In the bottom-up nanoparticle synthesis method, chemical and biological processes are exploited for NPs synthesis in the bottom-up nanoparticle synthesis method (Dahoumane et al. 2017). While the previous synthetic procedures involved ecologically harmful chemical agents, toxic by-products can also be created using environmentally corrosive compounds. Because of their highly specialized cells and tissue interaction capabilities and excellent efficacy in combating diseases, researchers are increasingly interested in using NPs in biomedical research (Sharifi-Rad et al. 2021).

Furthermore, plant-derived nanoparticles have emerged as novel treatment approaches for various disorders. Also, natural products, mainly plant extracts, contain insulin-sensitizing, anti-inflammatory, and antioxidant characteristics and are also regarded as a viable alternative due to their low risk of side effects (McCracken et al. 2018). This new green nanotechnology can now be utilized to generalize plant-mediated green synthesis of metallic nanoparticles, which is a significant step forward in the green synthesis of metal nanoparticles. Some metal-based nanoparticles have been discovered to have antibacterial capabilities and could be used as innovative materials to prevent the spread of antimicrobial resistance. The chemical composition, size, and form of nanoparticles are all being studied further to improve their synthesis. Two vital contributing aspects of nanoparticles’ physiochemical characteristics are increased particle size and the surface area-to-volume ratio entering the zone where quantum effects prevail (Elangovan et al. 2015; Vimbela et al. 2017).

Antibiotic resistance has grown to be a significant global public health issue. This is made worse by the lack of new medications, the development of resistance mechanisms in most clinical isolates of bacteria, and recurrent infections, which reduce the effectiveness of disease treatment (Adeniji et al. 2022). However, Nanoparticles (NPs) are increasingly used to target bacteria as an alternative to antibiotics. Pathogenic microorganisms have been combated using medicinal herbs and nanosilver. Herbal medications are commonly used in healthcare due to their inexpensive cost and abundance of antibacterial qualities (Okeke et al. 2021; Enechi et al. 2022). Silver nanoparticles, like medicinal plants, are finding new applications in biomedical sectors due to their inherent therapeutic properties and the reaction of Gram-negative and Gram-positive bacteria to different plant parts, including bark, stem, leaf, fruit, and seed, using other extraction solvents like methanol, ethyl acetate, chloroform, acetone, *n*. hexane, butanol, petroleum ether, and benzene (Ezeorba et al. 2022; Chukwuma et al. 2023a). Herbal medicines have been utilized in most countries since ancient times. Still, medicinal plants in Asia are extensively employed as a therapy for infectious diseases in rural and backward areas (Ezeorba et al. 2021; Ezema et al. 2022; Omeje et al. 2023). However, little research has elucidated the mechanism of action of plant-derived nanomaterials on antimicrobial resistance and metabolic disorders. The specific antibacterial processes of NPs have yet to be fully understood, and different types of NPs generally have different effects (Wang et al. 2017).

Nevertheless, the antibacterial mechanism of action of NPs can be classified into three categories: oxidative stress induction, metal ion release, and nonoxidative mechanisms (Khan et al. 2019). These three types of mechanisms can all happen at the same time. According to the recent research, Ag NPs cause the bacterial membrane's surface electric charge to be neutralized and its penetrability to alter, resulting in bacterial mortality (Hu et al. 2022).

Based on the foregoing, the review explores the antibacterial mechanisms of PDNMs, which can be categorized into oxidative stress induction, metal ion release, and nonoxidative mechanisms. It discusses the potential of PDNMs, such as Ag NPs, in targeting pathogenic microorganisms and combating antimicrobial resistance. Also, the use of plant-derived nanomaterials as drug carriers and their application in overcoming current challenges in antimicrobial resistance and metabolic diseases was elucidated. This study presented a classical review of recent findings on plant-derived nanomaterials with the potential for combating pathogenic bacteria, fungi, viruses, and other metabolic diseases. Relevant and recent works of literature were retrieved from Scopus, PubMed, and ScienceDirect repositories. Due to the current scientific interest in this subject, more attention and priority were given to recent studies published from 2015 to 2022.

## An overview of the method for preparation of plant-derived nanomaterial

**Fig. 1:**
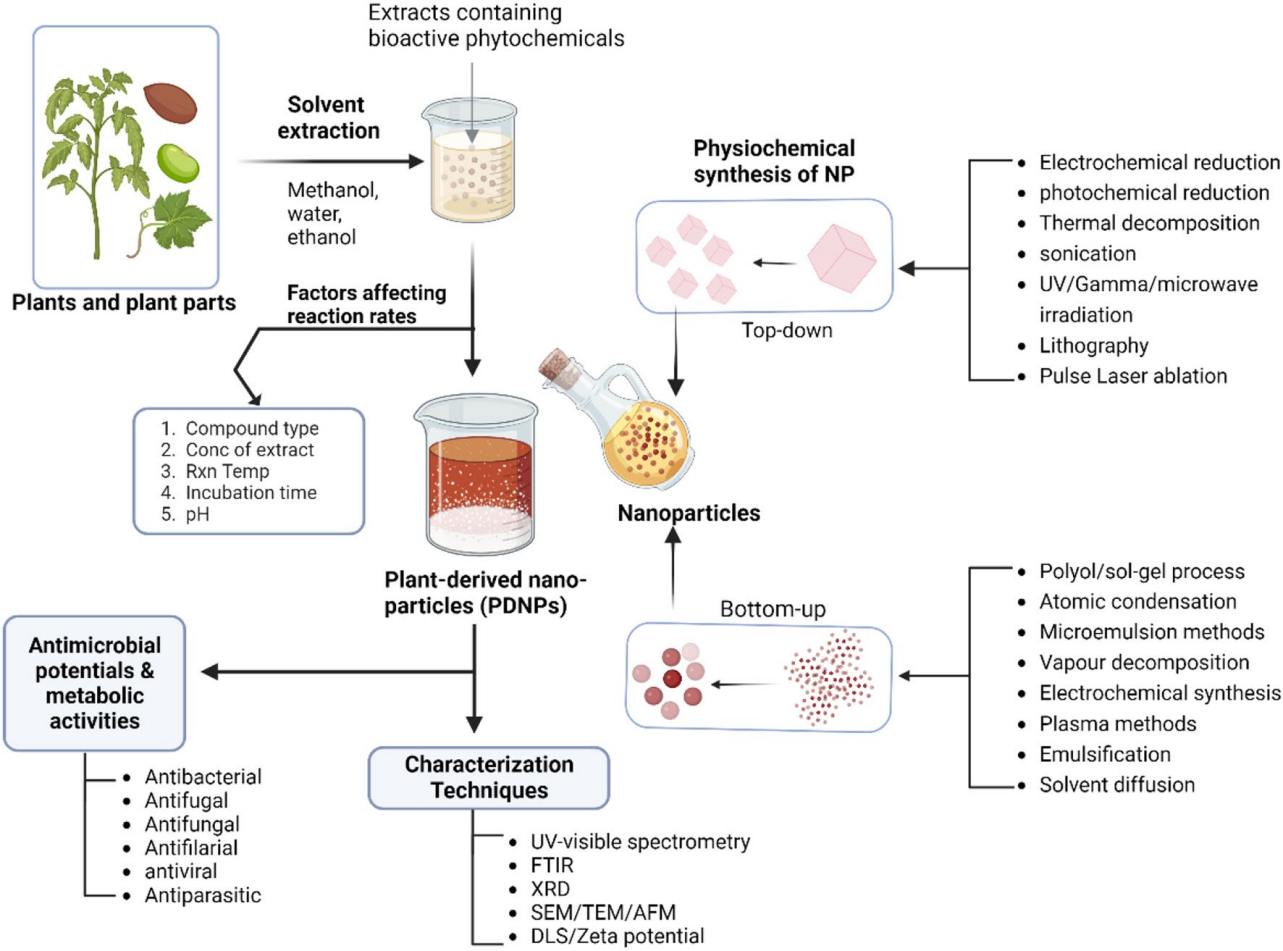
Methods for preparing plant-derived nanoparticles (PDNPs). Plant extracts from general solvent extraction are prepared with the synthesized nanoparticles to yield PDNPs. Factors affecting the formation rate of PDNPs include the phytocompound types, extract concentration, reaction temperature, incubation time, and others. The PDNPs are generally characterized and analyzed for bioactivities. The general physiochemical methods for preparing the nanomaterials have been categorized as top-down or bottom-up methods. The top-down methods begin with a macro particle which is then processed into smaller bits of nanoparticles. In contrast, the bottom-up involves the growth of subionic/pico-sized species to nano-particles

The synthesis of plant-based nanoparticles in human therapeutics has gained more attention in nanotechnology due to its effectiveness and eco-friendly acceptability. The eco-friendly nature of nano-particles synthesis is the building block in the plant-based nano-particles arsenal in fighting and eradicating various diseases (Villaseñor-Basulto et al. 2019). The major biosynthesis approach in nanoparticles is top-down and bottom-up. The unique thing about these methods is that they are synthesized by applying chemical, physical, and biological processes (Zhang et al. 2016b). The advantages of plant-based nanoparticle synthesis include using aqueous solvents, plant material availability, and biocompatibility. Plant secondary metabolite extracts are the target components for green nanoparticle synthesis in medical therapeutics. Its relevance in nano drugs development has opened a new era in emerging medicine (Fig. 1) (Mohammadinejad et al. 2015; Hano and Abbasi 2022).

Zinc, gold, silver, nickel, iron, platinum, and selenium are metals of choice in the studies of nanoparticle synthesis. Of all these metals, gold nanoparticles (AuNPs) are widely applied in biomedicine and imaging. AuNPs can be synthesized easily and have been proven less toxic than other metals. The size of AuNPs is smaller than that of nanoparticles synthesized from other metals and, thus, passes through the cell membranes faster. This may likely be why the potent antimicrobial, cytotoxicity, and anti-inflammatory actions exhibited (Can 2020; Keskin et al. 2022).

The significant property of nanomaterials is their shape. It has been reported that nanoparticles with spherical shapes pass through the cell membrane quickly to their target cells (Peralta-Videa et al. 2016). However, the surface charge (zeta potential) is considered at shallow doses of plant-based nanoparticles. The zeta potential of nanomaterials in pharmacology is vast. It determines the stability of nanoparticles through their release at their point of action and directs their movement in the bloodstream toward the body membrane for their absorption. Nanoparticles with negative zeta potential possess higher cellular uptake than positive ones. The lower the negative charge of nanoparticles, the easier the passage into the cell to achieve better interaction with the cellular membranes (Mohammadinejad et al. 2015; Hanna and Saad 2020; Hano and Abbasi 2022). This gives nano-particles the advantage in pathogenic microbes and cancer cell studies.

### Chemical methods

Several chemical approaches to synthesizing PDNM include polyol, microemulsion, and thermal decomposition. The polyol method utilizes nonaqueous liquid as a solvent and reducing agent. This solvent has the advantage of reducing surface oxidation and clustering. It permits nanomaterials' size, texture, and shape controls during production and favors the large-scale production of nanoparticles. The polyol method is sometimes called a sol–gel method in metal oxide synthesis if the temperature increases and particle growth is controlled. Owing to its high reducing capacity and boiling point, ethylene glycol is the preferred solvent in the polyol method. It plays a vital role in metal oxide ion oligomerization (Cele 2020; Trivedi et al. 2022b).

Similarly, the microemulsions method uses oil and water immiscibility flux to generate the energy needed for production. The interfacial tension between the two liquids is very high and can be overcome using surfactants. The preparative procedure of this method involves the mixture of two microemulsions embedded with metal salt and a reducing agent. The formation of Brownian motion occurs after the mix of the two microemulsions. A good collision is an indicator of a better reactant formation. The size and shape of nanoparticles formed are determined by the size and shape of the nanodroplets and the nature of the surfactants used. The surfactant provides stability and protection to the particle (Peralta-Videa et al. 2016; Cele 2020). Iron (III) oxide nanoparticle is formed by combining adequate water in a stock solution of Sodium Bis (2-E Ethylhexyl) Sulfosuccinate in *n*-heptane and left overnight. Afterward, concentrated hydroxylamine and ferric chloride were mixed into it. Iron (III) oxide suspension was filtered, washed with 95% ethanol, and dried at 300 °C for 3 h. The spherical product has a diameter of about 50 nm (Cele 2020).

Thermal decomposition (Thermolysis) is a chemical decomposition that requires heat to break the chemical bond of compounds involved in decomposition. Depending on the combustion heat applied, the reaction could be endothermic or exothermic (Patil et al. 2002; Moghaddam et al. 2022).

Electrochemical synthesis of plant-derived nanomaterials uses electrochemical cells to dissolve a metallic anode in the solvent. Electrochemical synthesis has been used to produce silver nanoparticles through the electroreduction of silver ions anode of acetonitrile in the presence of tetrabutylammonium. The intensity of the current density determines the size of the particle obtained. This method has been used to produce silver nanoparticles with fewer impurities. The production step is less expensive with controlled temperature and does not utilize dangerous chemicals. The silver nanoparticles’ shape is spherical, and their particle size is less than 50 nm (Lingaraju et al. 2016; Cele 2020).

### Physical methods

Some physical methods of preparing PDNM are plasma, vapor deposition, Microwave irradiation, Pulse laser, and Gamma radiation. In the plasma method, the radio frequency heating coils generate the plasma. This method operates in an enclosed metal inside a pestle which is also held in an evacuated chamber. It works by heating the fz metal above its evaporation point using high-voltage coils strapped around the evacuation chamber. The gas used for heat generation in this process is Helium. The Helium gas also diffuses the vapor from the metal up to the point of its cold collector rod, which serves as the point of the nano-particles collection after undergoing oxygen gas passivation (Can 2020; Hano and Abbasi 2022).

The physical vapor deposition (PVD) method involves a physical reaction and volatile precursors exposed to the substrate to form the desired deposit. Once the precursors are vaporized, they are inserted into a CVD reactor and, at high temperatures, adsorbed on a substance. The adsorbed molecules either react with other molecules or form crystals after decomposing. The advantage of this method over other chemical methods is that it synthesizes particles less than 1 μm (Peralta-Videa et al. 2016; Cele 2020).

The Microwave irradiation method is widely preferred in organic, inorganic, and hybrid organic–inorganic materials synthesis. This is because it facilitates reaction rate, improves purity and higher yield, and is eco-friendly as compared to conventional methods. Silver nanoparticles (AgNPs) have been synthesized using microwave-assisted irradiation using hexamine and biopolymer pectin as the reducing agent and stabilizer. Characterizing the synthesized AgNPs utilized UV–VIS spectroscopy, X-ray energy dispersive and diffraction, and transmission electron microscopy. The shape of AgNPs formed is spherical, with an average diameter of 18.84 nm (Peralta-Videa et al. 2016; Zhang et al. 2016a, b, c).

The Pulse laser method is widely used in silver nanoparticle synthesis and records a higher production rate of 3 g/min. The production step involves pouring silver nitrate solution and a reducing agent into a device that is blender-like in function. The device has a solid blade (disc) that rotates with the solution and exposes the edge to pulses from the beam, thereby creating hot spots on its (disc) surface. The hot spots are the reaction point of silver nitrate with a reducing agent to form silver particles. These particles can further be separated by centrifugation. The particles' size depends on the energy applied and the angular velocity of the blade (Can 2020; Cele 2020).

The Sonochemical reduction method is helpful in ionic species nanoparticle synthesis of Mno^4−^, Pd^2+^, Au^3+^, and many other metals and their oxides. The reduction rates need to be controlled in the presence of citric acid, which acts as an organic stabilizer, to prevent the shape and size of metal particles formed. In platinum nanoparticle synthesis, methanol and ethanol solvents are utilized. At the same time, propanol serves as the reducing agent in the presence of chitosan and polyethylene glycol as the capping polymers to produce a nanoparticle size of approximately 3 nm [21, 25].

Gamma radiation is the preferred method of metal nanoparticle production due to its reproducibility, ease of controlling the shape, cheap operation, less toxic precursors, fewer reagents involved, controlled temperature, less waste generated, and few production steps. The Gamma radiation method works by excitation effects of the interaction of the metal ions solution causing an ionization action on the solvent. This method is applied mainly in gold and silver nanoparticle synthesis and is further characterized by UV–VIS spectroscopy (Cele 2020).

## General characteristics and properties of PDNM

### Physical characteristics of plant-based nanomaterials

**Fig. 2:**
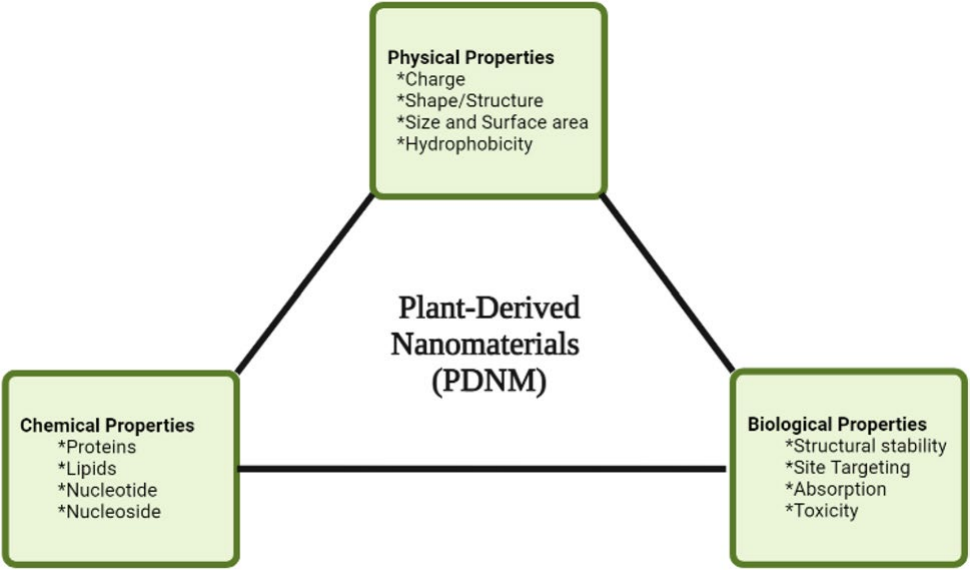
Schematic and summarized properties of plant-derived nanomaterials, categorized into the physical, chemical, and biological properties

The physical properties of PBNM, such as the size, shape, surface charge, and surface coating, play crucial roles in the potency of these nanomaterials. They are pertinent to the absorption rate, the mode of absorption, and cellular toxicity (Fig. 2).

#### Nanomaterial size and surface area

The sizes of nanomaterials and their surface areas are vital as they play essential roles in the interactions and relationship between these materials and the biological components or organization (Mourdikoudis et al. 2018). These interactions range from how these nanomaterials are absorbed, transported/distributed, and eradicated. Considering the size of these nanoparticles, their mode of uptake can be phagocytosis or pinocytosis. Nanomaterials with higher molecular weight (250 nm to 3 µm) will be absorbed via phagocytosis, while those with smaller weights can either be by phagocytosis or pinocytosis (Sun et al. 2020). Furthermore, phagocytizing heavy molecular weight nanomaterials are internalized with a gigantic phagocytic ability (Sun et al. 2020). Because one of the significant aims of exploring nanoparticles is to elongate their stay within the biological system, which indirectly improves their potency, increasing the sizes may escalate the rate at which it is eliminated from the physical system. Therefore it has become pertinent to observe and monitor their measures for appropriate application (Foroozandeh and Aziz 2018). The average length of some investigated plant-based nanomaterials has ranged from 0.11 to 344.9 nm (Hesari et al. 2021). Nanoparticles from ginger had sizes of 220–290 nm (Wang et al. 2013), and those derived from grapefruits were 180–200 nm (Teng et al. 2016). Some findings suggested that the scope for optimum cellular uptake was within 50 nm and decreased when it was < 30 nm and > 70 nm. These findings are inconsistent as some other studies have shown varying sizes (outside the former ranges) achieving optimum internalization (Foroozandeh and Aziz 2018). These sizes can be managed in a novel strategy described by the work done by Zhang et al. (2016a, b, c). They can also be altered via temperature changes, alterations in the buffer system, and pH. pH can either increase or decrease the sizes of these plant-derived nanoparticles (PDNM and this may be dependent on the plant source (Mu et al. 2014).

#### Shape or structure

The internalization of nanoparticles is also largely dependent on the shape or design of these nanoparticles. The most natural shape is spherical as they are more internalized when as compared to other conditions. The common NP shapes come in different forms, such as spherical, rod-like, disc-shaped, and cylindrical forms (Banerjee et al. 2016). The PDNM has similar structures to the biological cells and is also spherical. The lipid-bilayer in PDNM limits the diversity of the structures that can be constructed to suit their various purposes and enhance their potency. The studies and findings can back this up when nanoparticles derived from grapefruit were reconstructed into flowery forms to improve their potency (Wang et al. 2015).

#### Charge

The charge possessed by PDNM plays a vital role in the relationship between them and the biological host system (Table 1). They remarkably affect the biological components as they govern their interactions (Sun et al. 2020). Cells with membrane-bound nuclei (Eukaryotic) naturally are bestowed with negative charges and maintain a membrane potential of − 40 mV to − 80 mV. The costs seen in PDNM are also negatively charged, which is expected as they are usually derived from eukaryotic cells. The charges on synthetic nanomaterials (NM) facilitate their attachment and uptake within the biological system (Foroozandeh and Aziz 2018). As elaborated by the laws of physics with charges, positively charged NM will be absorbed or internalized faster since the cell membranes are negatively charged. However, the absorption of positively charged synthesized NM may lead to toxicity and cell death via the destabilization of membrane integrity (causing cell fluidity). In contrast, the uptake of neutral PDNM may cause the gelation of membranes.

**Table 1:** Physical properties of some PDNMs

Source	PDNM	Size (nm)	Charge (m/V)	References
Curcumin	Curcumin nanoemulsion	42.93 ± 29.8	− 0.12 ± 0.50	Rachmawati et al. (2016)
Cherry	Quaternary ammonium chitosan Cherry extract loaded NPs	344.9 ± 17.8	14.8 ± 0.3	Beconcini et al. (2018)
Cherry	S-protected thiolated derivative	339.9 ± 68.2	15.8 ± 0.5	Beconcini et al. (2018)
Curcumin	liposomes loaded with atorvastatin calcium and curcumin	192 ± 0.8	6.78 ± 0.99	Li et al. (2019b)
Curcumin	Curcumin- poly(ethylene glycol) methyl ether-block-poly(D,L- lactide	~ 50	− 14.9	Li et al. (2017a)
Tanshinone IIA	Tanshinone IIA nanoparticles	100–200	-7.12 ± 0.07	Mao et al. (2018)
Resveratrol	Resveratrol-solid lipid nanoparticles	~ 271.13	− 25.8 ± 0.33	Zhang et al. (2019)
Quercetin	Quercetin-loaded poly lactic-co-glycolide	165 ± 75	− 28 8 ± 12	Guan et al. (2016)
Baicalin	baicalin-loaded PEGylated nanostructured lipid carriers	83.9 ± 1.6	− 32.1 ± 1.8	Lozano et al. (2019)
Ginsenoside Rg3	Rg3-loaded Pluronic	49.44 ± 0.15	NA	Li et al. (2017b)
Puerarin	RGD-modified and PEGylated solid lipid nanoparticles loaded with puerarin	110.5 ± 3.4	− 26.2 ± 1.8	Dong et al. (2017)
Resveratrol	RSV-NC	207 ± 0.03	− 7.15 ± 0.19	Shahraki et al. (2017)
Puerarin	puerarin-loaded 1,2-distearoyl-sn-glycero-3-phosphoethanolamine-N-[methoxy(polyethylene glycol)-2000]	~ 17.1	− 6.24	Li et al. (2019a)
Breviscapine	breviscapine lipid emulsion	225.3 ± 8.8	− 29.6 ± 1.5	Xiong et al. (2010)
Magnolol	Magnolol Nanoparticles	75.6 ± 1.7	NA	Lee et al. (2018)
Curcumin	curcumin encapsulated by carboxymethyl chitosan (CMC) nanoparticle conjugated to a myocyte-specific homing peptide	331.2	+ 11.2	Ray et al. (2016)

#### Hydrophobicity

The hydrophobicity of PBDM informs us how they will be internalized. Some may be internalized within the hydrophobic cores of the cell membranes (hydrophobic nanomaterial), while some will be wrapped around (Foroozandeh and Aziz 2018).

### Chemical properties of plant-derived nanomaterials

The composition of PDNM varies from those emanating from mammals. Plant-based nanomaterials are more flexible because they have lower concentrations of phospholipids with little or no cholesterol when juxtaposed with those from mammals (Stremersch et al. 2016). Their lipid and protein concentrations also vary.

#### Proteins

Proteins on the surface of these plant-derived nanomaterials contribute immensely to intracellular interactions. It is believed that proteins seen in plants are not usually difficult to fabricate during nanomaterial formation as compared to mammalian-derived nanomaterials, as they are not as ingenious as mammals (Vader et al. 2016). A few findings have elaborated on the concentration and composition of the proteins found in PDNM. Some studies on ginger-derived nanomaterials have been found to contain proteins such as actins and proteolytic enzymes (cytosolic) in higher amounts than some membrane proteins (Zhang et al. 2016a). These proteins also bestow immensely on the structures of PDNM. However, those derived from lemon juice showed high protein content and can also be associated with those derived from mammalian cells (Yang et al. 2018).

#### Lipids

This is an essential part of the bilayers of plant-derived nanomaterials. They vary from those derived from the exosomes of mammalian and other synthetic origins (Sheikhpour et al. 2017). The exosomes or vesicles on mammals’ nanomaterials have a high phosphatidylcholine content and about 10–20% cholesterol (Ha et al. 2016; Yang et al. 2018). These lipids play a significant role in stabilizing and strengthening the walls of these materials. Some research findings showed that nanomaterials of plant origin (ginger) were devoid of cholesterol, while those containing cholesterol had a different configuration from nanomaterials derived from mammals (Mu et al. 2014).

#### Nucleosides and nucleotides

Nucleosides such as ribonucleic acids (RNA) can be transferred from parent to offspring or incorporated into new cells with foreign vesicles. This can be found in some plant and mammalian vesicles where RNAs such as miRNAs and lincRNAs are incorporated into new or receiving cells. Some PDNM also contains miRNAs to improve interactions between the host cells and increase their potency. Some PDNM has been found to contain hundreds of RNAs and nucleotides that are below 30 in number. In silico findings have also suggested that PDNMs containing some miRNAs may have interspecies links at the molecular level (Yang et al. 2018).

### Biological properties

The biological properties of plant-derived nanoparticles will be viewed under structural stability, site targeting, toxicity, and adsorption.

#### Structural stability

Most synthetic nanoparticles are more stable than plant-derived nanoparticles. Their properties are also more predictable than the PDNM. However, this stability can increase the risk of toxicity to the targeted cells. This toxicity may be a result of alterations done artificially to improve its ability to reach its target cells and then metabolize to yield products that may be toxic. The PDNM, on the contrary, is absorbed rather than metabolized since its structural properties are made of natural components such as lipids. Nonetheless, some findings oppose the idea that PDNMs are less stable than synthetic nanomaterials (Yang et al. 2018). This was seen in some ginger-derived nanomaterials that were very stable in solutions that mimicked that of the stomach and the small intestines as they resisted disruption in repeated freeze and thaw cycles (Zhang et al. 2016a).

#### Site targeting

One of the primary goals of nanoparticles is to bind at the targeted sites. They must be designed to cluster within the target of choice. This has made tissue organization and arrangement studies pertinent to creating nanoparticles (Kim and Kim 2017). Some findings have suggested that different routes of exposure to these nanoparticles also lead to different localizations and elimination rates. Grapefruit nanoparticles that were localized in the brain took longer to be eliminated than those in the lungs. Plant-derived nanomaterials may demonstrate a good and safe passage through the placenta of pregnant mice over synthetic nanoparticles as the efflux transporters present in the placenta walls can recognize them (Goasdoué et al. 2017).

#### Absorption

The absorption of nanoparticles at the targeted sites is essential when designing nanoparticles. Designing them as a delivery tool is paramount in drug delivery systems. This absorption can be affected by some factors, such as the physiology of the target site, the size of the nanoparticles, and their interaction within the environment of the targeted system (Pérez-de-Luque 2017). Some plant-derived nanoparticles were treated, and the uptake was within 14 to > 20% of the T-cells and B-cells (Zhang et al. 2016b). Some synthetic nanoparticles have not achieved this. Some findings have also shown an uptake of above 80%, while some synthetic nanoparticles had just about 40% (Yang et al. 2018).

#### Toxicity

One of the significant challenges facing synthesized nanoparticles is their toxicity. Due to varying modifications done on the synthetic nanoparticles to achieve the goal of reaching their target sites and being absorbed, it has indirectly increased its toxicity. These alterations increase the rate at which the immune system recognizes them as foreign and the rate at which it eliminates them from the system. This is an advantage of PDNPs over synthetic nanoparticles since they do not elicit immune responses on entry into the target system. The findings by Zhang et al. [32] where he used applied ginger-derived nanoparticles on some epithelial and macrophage cell lines and found them to be nontoxic.

## Therapeutic applications of plant-derived nanomaterials

### Antibacterial activities

Due to the steady rise of antibiotic resistance cases, scientists constantly seek alternative antibacterial options with improved efficacies and reduced toxicity or side reactions. Plant-derived nanoparticles have been reported to have gained applicability as antibacterial agents in recent years.

**Fig. 3:**
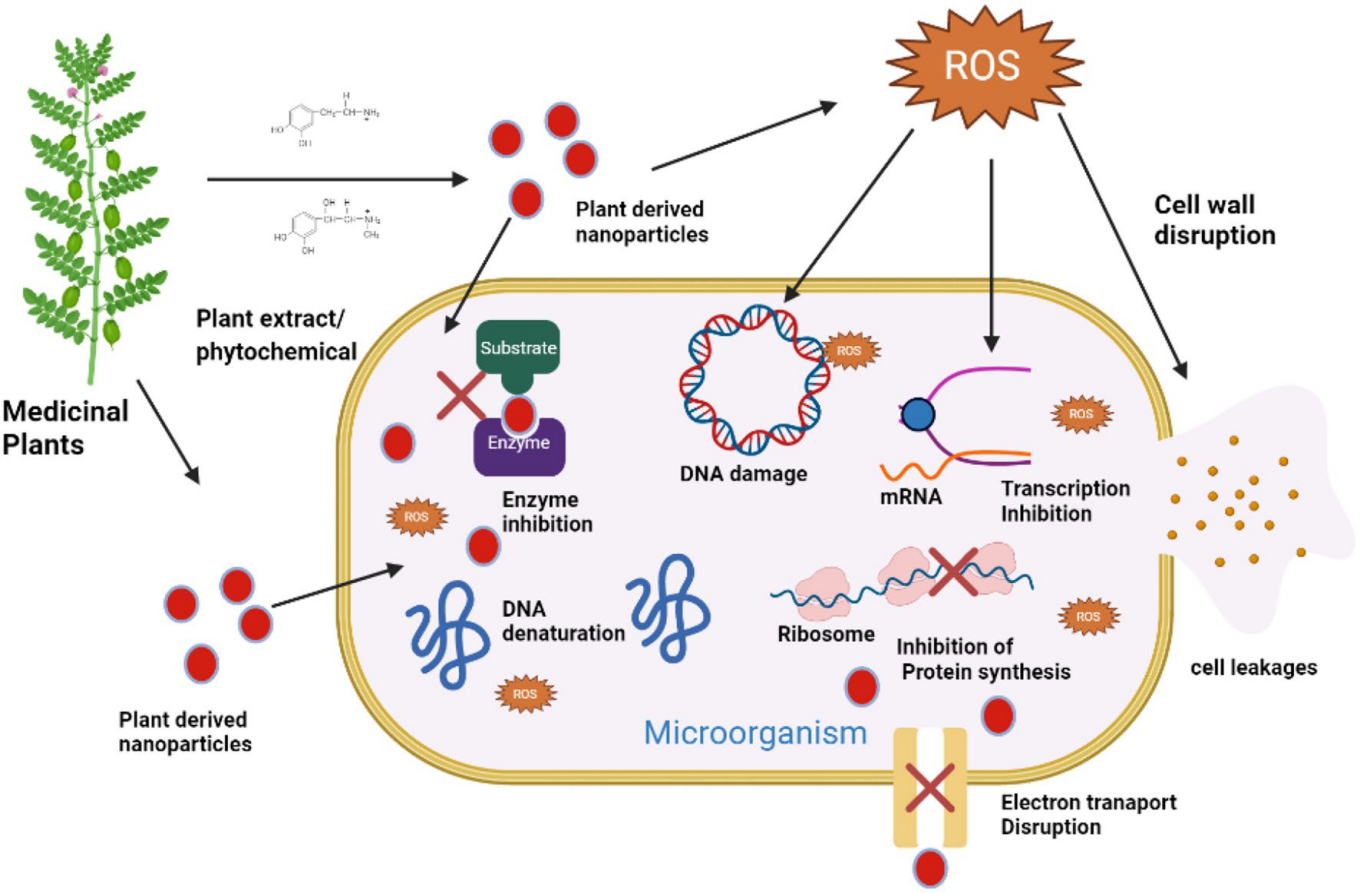
Mechanisms of antimicrobial activities of plant-derived nanoparticles (PDNPs). PDNPs prepared from plant extracts have been to exhibit several mechanisms which promote the anhiliation of different microbial species. PDNPs could enter the cells, causing DNA denaturation, DNA damage, inhibtion of transcription and protein synthesis inhibition, and inhaling valuable enzymes for virulences or microbial cell survival. Moreover, PDNP can also promote the generation of oxidative species (ROS), damaging the genetic constituents of microbial cells and could cause apoptosis or cell leakages

Generally, the mechanism of bactericidal effects of PDNP is either extracellular or intracellular. The PDNPs can accumulate on bacterial surfaces, inhibiting favorable signal inductions and cellular transport. On the other hand, PDNP may gain entry into the microbial cytoplasm, which then triggers the nanoparticle release and alters bacterial activities. The charge on the PDNP determines the effect on specific bacterial strains (Fig. 3). Banasiuk (Banasiuk et al. 2020) recently reported that positively charged green synthesized silver nanoparticles (AgNPs) were more effective against negatively charged bacterial cells. Other studies have reported the ability of PDNP to generate ROS as an alternative mechanism for their antibacterial inhibitory activities. Properly characterized, rice-shaped copper oxide nanoparticles, synthesized from *Caesalpinia bonducella* plant extracts, were very effective against gram-positive *Staphylococcus aureus* and gram-negative *Aeromonas* via an in vitro agar diffusion method. This study also reported the membrane disruption ability of the *Caesapinia bonducella nano*particles on both bacteria strains (Sukumar et al. 2020).

Another study has reported the antibacterial potency of iron nanoparticles prepared from *Hibiscus rosa sinensis* flower extract and FeCl_3_6H_2_O solution in the ratio of 1:1 against several bacteria strains such as *Staphylococcus aureus, Klebsiella pneumonia, Salmonella typhi*, and *Pseudomonas aeruginosa.* The Iron-nanoparticles synergistically with the plant extract foster the generation of free radicals that disrupt the membrane permeability of microorganisms (Buarki et al. 2022). Finally, similar studies have fabricated Nickel nanoparticles from *Stevia rebaudiana* extract and characterized them via several assays such as XRD, TEM/SEM, and X-ray photoelectron spectroscopy (XPS). The PDNPs from *Stevia* showed tremendous antioxidant and antimicrobial activities against gram-negative *E. coli* and gram-positive strains of *Bacillus* and *Streptococcus* (Srihasam et al. 2020).

A few studies have tried using characterized and purified compounds (existing naturally in plant extract or chemically synthesized) for fabricating nanoparticles. Natural flavonoids such as s quercetin pentaphosphide (QPP), quercetin sulphonic acid (QSA), and apigenin triphosphate (ATRP) have successfully been used to develop silver nanoparticles with tremendous antibacterial activities against *Staphylococcus epidermidis, E. coli*, and *Citrobacter freundii* ATCC 8990 (Osonga et al. 2016). Moreover, in a study by Mousavi-Khattat et al. (2018), the stability, antioxidant, DNA cleavage, and antibacterial activities of plant-derived silver nanoparticle was as compared to their chemically synthesized counterpart. It was reported that the green synthesized NPs from *Datura stramonium* leaf extract showed more desirable qualities such as a more narrow nanoparticle and spherical shape, high antioxidant, better antibacterial, and DNA cleavage activities than the synthetic nanoparticles. In short, the synthetic nanoparticle showed zero antioxidant capacity and insignificant antimicrobial and DNA cleavage activities (Mousavi-Khattat et al. 2018). Other reports on the antimicrobial properties of PDNP are presented in Table 2

**Table 2:** Pharmacological potentials of Plant-Derived Nanomaterials against pathogenic microbes and metabolic conditions

Biological Activities	Plant	Plant part	Nanomaterials	Shape	Sizes (nm)	Activities	References
Antibacterial	*Brassica oleracea*	leaf	AgNPs	spherical	Average 20 nm	Significant antibacterial zone of inhibition (9-14 mm) on microbes such as *Bacteroides fragilis and Staphylococcus epidermidis, Pseudomonas aeruginosa, Enterococcus faecalis and Proteus mirabilis, Klebsiella pneumoniae*	Ansar et al. (2020)
	*Cassia auriculata*can	leaf	ZnO Nps	spherical	Average 30 nm	Strong zone of inhibition between 8.4 and 40.2 mm at a conc of 200 μg/ml *Bacillus subtilis, Klebsiella pneumonia, Pseudomonas aeruginosa.* and *Proteus mirabilis*	Ramesh et al. (2021)
	Grapefruit	Peel (Ethanol and aqueous extract)	AgNPs	Not specified	160.5 nm	*S. aureus, E. faecalis and E. coli* at an MIC of 1.5625—12.5 mg/mL and MBCs of 6.25–50 mg/mL	Arsène et al. (2021)
	*Citrus limon*	peel	AgNPs	spherical	Average 59.74 nm	Zone of inhibition of 35 mm in *E. coli* and *S. aureus*	Alkhulaifi et al. (2020)
	*Premna integrifolia*	leave	AgNPs	Spherical	9–35 nm	*Antibacterial effect on Staphylococcus aureus*, *Enterococcus faecalis, Shigella dysenteriae*, *Shigella flexneri*, and *Vibrio parahaemolyticus* at a MIC of 16.5–66 µg/mL	Singh et al. (2019b)
	Coffee	Seed residue	AgNPs	Spherical	50 nm	Completely inhibit the growth of *E.coli* at a concentration of 200 μg/ml after 12 h	Baghaienezhad et al. (2020)
	*Artocarpus integer*	leaf	AgNPs	Spherical	5.76–19. 6 nm	Zone of inhibition of 14–29 mm for *Staphylococcus aureus, Bacillus cereus, Salmonella entertica* and *Escherichia* *coli* at a NP conc of 12 µg/ml	Majeed et al. (2019)
	*Anogeissus acuminata*	Leaf	AgNPs	Spherical	Below 100 nm	A zone of Inhibition (Z1) between 13- 19 mm of UTI bacteria at a conc of 15 μg/ml np	Mishra and Padhy (2018)
	*Syngonium podophyllum*	Leaf	AgNPs	Spherical	2 to 47 nm	At a conc of 6.25 to 25.00 µg/mL, about 70% MIC of *Escherichia coli, Pseudomonas aeruginosa, Bacillus subtilis*, and *Staphylococcus aureus,* was observed after 5 h	Naaz et al. (2021)
	*Malus domestica and Cuminum cyminum*	Pulp aqueous extract	AgNPs	spherical or globular	1.84–20.57 nm	Zone of inhibition of 9.9–12.53 mm against *S. aureus* and *E. coli*	Jahan et al. (2021)
	*Plantago major L*	Leaf	AgNPs	Spherical	10–20 nm	*Staphylococcus aureus, Escherichia coli*, and *Pseudomonas aeruginosa at a ZI of 5.76–9.91 mm at 20* µg mL^−1^ NP	Sukweenadhi et al. (2021)
	*Crescentia alata, Randia echinocarpa, and Vitex mollis*	Fruit melanin	AgNPs and AuNPs	Spherical	2–16 nm	MIC = 1.85–15 µg mL^−1^; MBC = 3.7–30 µg mL^−1^ against *Shigella dysenteriae*	Montes-Avila et al. (2022)
	*Ligustrum vulgare*	berries	AgNPs and AuNPs	More spherical-shaped nanoparticles, although a few detections of triangular, hexagonal rods; cuboid shapes particles	20–70 (AgNPs) and 50–200 nm (gold)	Annihilate *P. aeruginosa* and *E. coli* at 100 and 150 µg/mL, respectively, for AgNPs	Singh and Mijakovic (2022)
	*Berberis vulgaris*	Leaf and root	AgNPs	spherical	30–70 nm	The MIC 5 mM of *S. aureus* and *E. coli*	Behravan et al. (2019)
	*Celosia argentea*	Whole plant	Cobalt NPs	Spherical	Average size 27.42 nm	Zone of inhibition of *E. coli* (51.83 mm) and *B. subtilis* (42.18 mm)	Shahzadi et al. (2019)
	*Astragalus spinosus*	Whole plant	AgNPs	Spherical	30–40 nm	MIC values for *S. mutans* and *A. viscosus* were 10.6 and 13.3 μg/ml, respectively	Ghabban et al. (2022)
	*Myristica fragrans*	fruit	ZnO-NPs	the spherical or elliptical shape	41.23 nm	the inhibition against *Staphylococcus aureus* (21 ± 1.73 mm), *Escherichia coli* (15 ± 1.54 mm), *Klebsiella pneumonia* (27 ± 1.73 mm), and *Pseudomonas aeruginosa* (17 ± 1.66 mm)	Singh and Mijakovic (2022)
Antifungal	*Psidium guajava* or *Tamarindus indica*	leaf	AgNPs	Polyshaped morphology consists of spherical, triangular and plate-like	5–53 or 12–91	Significantly inhibit (p < 0.05) the growing rate of *Fusarium oxysporum, Aspergillus niger,* and *Aspergillus flavus*	Le et al. (2021)
	*Passiflora foetida*	Fruit	AgNPs	Spherical	Average 12 nm	Zone of inhibition of 22 ± 0.3 mm at 80 μg/mL of AgNPs against *Fusarium sp.*	Elangovan et al. (2022)
	Garcinia mangostana and radescantia spathacea	Shell and leaf	AgNPs	spherical	15.8 and 22.4, respectively	Significantly inhibits the proliferation of *Aspergillus niger, Aspergillus flavus, and Fusarium oxysporum*	Le et al. (2020)
	*Stachys lavandulifolia*	leaf	superparamagnetic iron oxide NP (SPIONP and AgNPs	`spherical	2.57 nm and 10.70 nm, respectively	Inhibitory activities against *Aspergillus niger* and *Fusarium solani*	Azhdari et al. (2020)
	*Justicia adhatoda*	leaf	Zinc Oxide NP	Spherical	11.6	Inhibits the growth of *Aspergillus niger, Aspergillus flavus*, and *Aspergillus fumigates*	Pachaiappan et al. (2021)
	*Dimocarpus longan*	fruit	AgNPs	Spherical and irregular	30–123	*C. ablicans, C. neoformans, A. niger growth* inhibition	Sathiya and Geetha (2021)
	Eryngium caucasicum	leaf	Ag/Fe3O4 nanocomposite	Spherical	26–42 nm	Highest inhibition of *Cryptococcus neoformans* at 150 μg/ml	Dehghan et al. (2021)
Antiviral	Olive leaves and natural honey	Leaf/ metabolite	ZnONPs and AgNPs	spherical and prismatic shapes	10–50 nm	Inhibit cytopathic effect of bovine herpesvirus-1 in MDBK cell culture at 25 μg/mL	Zeedan et al. (2020)
	*Oscillatoria* sp. and *Spirulina platensis*	Whole algae	AgO-NPs and Au-NPs	*Spherical (AgONP) *Octahedra, pentagonal, & triangular (Au-NPs)	14.42–77.13	90% reduction of cytopathic effect (CPE) and about 42.75–49.23% reduction rate of HSC-1 replication on Vero cells	El-Sheekh et al. (2020)
	*Panax ginseng*	root	Ultra-sonication preparation of AgNPs	spherical	5–15 nm	At a concentration of 0.25, about 15.12% inhibition of influenza A virus (strain A/PR/8/34) activities	Sreekanth et al. (2018)
	*Allium sativum (Garlic)*	Clove	AuNP	Spherical	6–11 nm	57% reduction of PFU of Measles virus at 10 μg/mL (EC_50_ of 8.829 μg/mL)	Meléndez-Villanueva et al. (2019)
	*Nigella sativa and Piper nigrum*	seeds	AgNPs	Spherical	20-50 nm	Decrease the viral load of HSV-1 to about 83.23—94.54%	Mahfouz et al. (2020)
Antitumor/ cytotoxic	*Cinnamomum tanala*	leaf	TiO_2_	irregular	23 nm	Exhibited a dose-dependent toxic effect on D145 cells	He et al. (2018)
	*Cynodon dactylon*	Leaf	TiO_2_	hexagonal and irregular	13–34 nm	Enhanced anticancer activity against A549 (lung cancer)	Hariharan (2019)
	*Costus pictus* leaf	leaf	MgO	spherical	50 nm	MgO NPs at 200 µg showed efficient anticancer activity	Suresh et al. (2018)
Antidiabetic	*Ananas comosus*	the outer peel of fruits	AgNPs	spherical	–	100% inhibitionofα-glucosidase	Das et al. (2019)
	*Myristica fragrans*	fruit	ZnO-NPs	the spherical or elliptical shape	41.23 nm	Significant inhibitory potential against enzymes protein kinase, α-amylase, α-glucosidase	Singh and Mijakovic (2022)
	*Saraca asoca*	Bark	AgNPs	spherical	< 5 nm	Significant antidiabetic and wound-healing property	Bairagi and Nath (2021)
Antioxidants	*Ananas comosus*	the outer peel of the fruit	AgNPs	spherical	–	Moderate ABTS, reducing, and NOXs scavenging activities	Das et al. (2019)
	*Myristica fragrans*	fruit	ZnO-NPs	the spherical or elliptical shape	41.23 nm	Excellent antioxidant activity	
Antispasmodic	*Myristica fragrans*	fruit	ZnO-NPs	the spherical or elliptical shape	41.23 nm	larvicidal activity (77.3 ± 1.8) against *Aedes aegypti*	Singh and Mijakovic (2022)
Anti-inflammatory	Juglans regia	fruit	FeNPs	–	21 nm	significant anti-inflammatory activity and exhibited membrane stabilization of erythrocyte membrane in comparison with standard drug	Suresh et al. (2018)
	green tea	leaf	ZnO NPs	–	–	ZnO NPs significantly inhibited monosodium glutamate-induced oxidative stress and inflammation	Al-Salmi et al. (2019)
	Solanum lycopersicum	fruit	CeNPs	spherical	5–10 nm	maximum inhibition of protein denaturation	Pujar et al. (2018)
	*Saraca asoca*	Bark	AgNPs	spherical	< 5 nm	Significant anti-inflammatory property	Bairagi and Nath (2021)

### Antifungal activities

Plant-derived nanoparticles have also been reported to inhibit the growth of fungi, especially pathogenic ones. The antifungal mechanism of actions of PDNP is through the disruption of the fungi extracellular membrane by electro-statistic interaction of nano-ions as well as reactive oxygen species. Other mechanisms are the initiation of apoptosis by fostering mitochondrial oxidative stress, inhibiting ATP synthesis, and different signaling pathways. Previous and recent studies have identified potent antifungal activities in several PDNPs (Table 2). Silver nanoparticle fabricated from the leaf extract of *Brassica rapa* was reported to have tremendous potency against several multicellular fungi such as *G. trabeum, G. abietinum, C. globosum*, and *P. sordida*. (Narayanan and Park 2014). A similar study on silver nanoparticles from *Tropaeolum majus L* leaf extract effectively against well-known fungi and yeast pathogens such as *Candida albicans, Aspergillus niger, Trichoderma viridiae, Penicillium notatum*, *Mucor* sp., with a maximum MIC activity against *P. nodatum* at 31.2 μg/ml. (Valsalam et al. 2019). A more recent study also on silver nanoparticles from *Ligustrum lucidum* was effective against *Setosphaeria turcica *at an IC_50_ of 170.20 μg/mL (Huang et al. 2020). Other studies have tried other nanoparticles apart from well-popularized AGNPs. Also, in the study by Srihasam et al. (2020), Nickel nano-particles from Stevia were effective against different pathogenic strains of Aspergillus. Iron nanoparticles from *Crocus sativus* showed antifungal activities against wilts, causing fungi (*Verticillium dahlia*) in many crops (Alam et al. 2019). Studies have tried other plants extract for synthesizing PDNPs with antifungal activities, such as *Passiflora foetida* fruits (Elangovan et al. 2022) and *Dimocarpus longan* fruit extract (Sathiya and Geetha 2021) against *Fusarium* sp., *C. albicans, C. neoformans,* and *A. niger.*

### Antiviral and antiparasitic activities

Several PDNP has been reported to possess antiviral properties by inhibiting and preventing virus replication. Viruses are causative agents for several invasive conditions, possibly leading to terrific mortality. Some antiviral assays are performed on various cell cultures like vero, MDBK, HeLa cells, and many more. The antiviral capacity is usually determined by the level of inhibition or reduction of the cytopathic effect (CPE) in the cell culture.

Zeedan et al. (2020) recently reported the antiviral properties of Zinc and silver nanoparticles synthesized from olive leaves and natural honey. The nanoparticle form had spherical and prismatic shapes between a nano range of 10–50 nm and was very effective at 25 μg/mL in inhibiting CPE in MDBK cells inoculated with bovine herpesvirus-1 (Zeedan et al. 2020). Similarly, a 90% reduction in CPE caused by HSV-1 in Vero cell culture was achieved by treating the cells with silver and gold nanoparticles of multi-shaped morphology from green algae (*Oscillatoria* sp. and *Spirulina platensis*). (El-Sheekh et al. 2020).

Other viruses, such as influenza A virus (strain A/PR/8/34), measle virus, and Japanese encephalitis virus, have also been susceptible to some PDNP (Sreekanth et al. 2018; Meléndez-Villanueva et al. 2019; Mehmood et al. 2020). An older study has reported using plant-purified metabolites, Catechin, for fabricating gold nanoparticles of an average size of 50 nm. The study showed the very excellent antiviral properties of the formulation by inhibiting 100% CPE caused by the Japanese encephalitis virus even at a very low concentration of 0.04 to 5.85 μg/ml (Chowdhury et al. 2016). This is, therefore, evidence that purified phytochemicals may be more productive than crude plant extracts for therapeutic purposes. Other studies showing the antiviral properties of plant-derived nanoparticles have been summarized in Table 2.

Plant-derived nanoparticles have also shown impressive potencies against other health-debilitating parasites and pathogens, including worms (helminth) such as filaria. Some interesting studies have shown the antifilarial activities of biogenic synthesized gold and silver nanoparticles against parasites such as *Setaria cervi* and *Wuchereria bancrofti* (Saini et al. 2016; Saha et al. 2017; Roy et al. 2018)*.* These biogenic nanoparticles were prepared in different formulations with and without nature-derived chitosan, as well as some plant extracts, including *Acacia auriculiformis funicles, Piper nigrum (black pepper), and Terminalia chebula* (Roy et al. 2018)*.* Generally, these nanoparticles were characterized through several protocols, including TEM, SAED, Z-potentials, UV spectroscopy, and others. They had an LC_50_ of 5.61 μg/mL for *Setaria cervi* and 4.3 μg/mL for *W. bancrofti*, respectively (Saha et al. 2017). Further experimental evidence showed that the nanoparticles fostered the annihilation of the parasites by inducing oxidative stress, promoting their apoptosis, and altering their Nrf2 signaling. Finally, despite the tremendous potencies, it was conclusively shown that the nanoparticles were nontoxic to the mammalian system (Roy et al. 2018).

Malaria has been a severe health disturbance, especially in African countries, due to its several mechanisms of evasion and acquisition of drug resistance (Ezema et al. 2023). Plant-derived nanomaterials have been a recent alternative to combat the health menace caused by the malaria parasite (Okagu et al. 2022). Several other recent studies have demonstrated the potencies of PDNM against other parasites such as plasmodium (Chukwuma et al. 2023b). Extract from a brown seaweed (*Sargassum wightii*) used in the biogenic synthesis of ZnO nanoparticles and characterized by XRD, SEM. EDX and FTIR were shown to possess interesting larvicidal and pupicidal activities against *Aedes stephensi *(with an LC50 of 12.278–20.798 ppm) (Murugan et al. 2018). More discussion on the antimalaria activities of green synthesized nanoparticles has been recently reviewed (Mohammadi et al. 2021).

### Antitumor activity

**Fig. 4:**
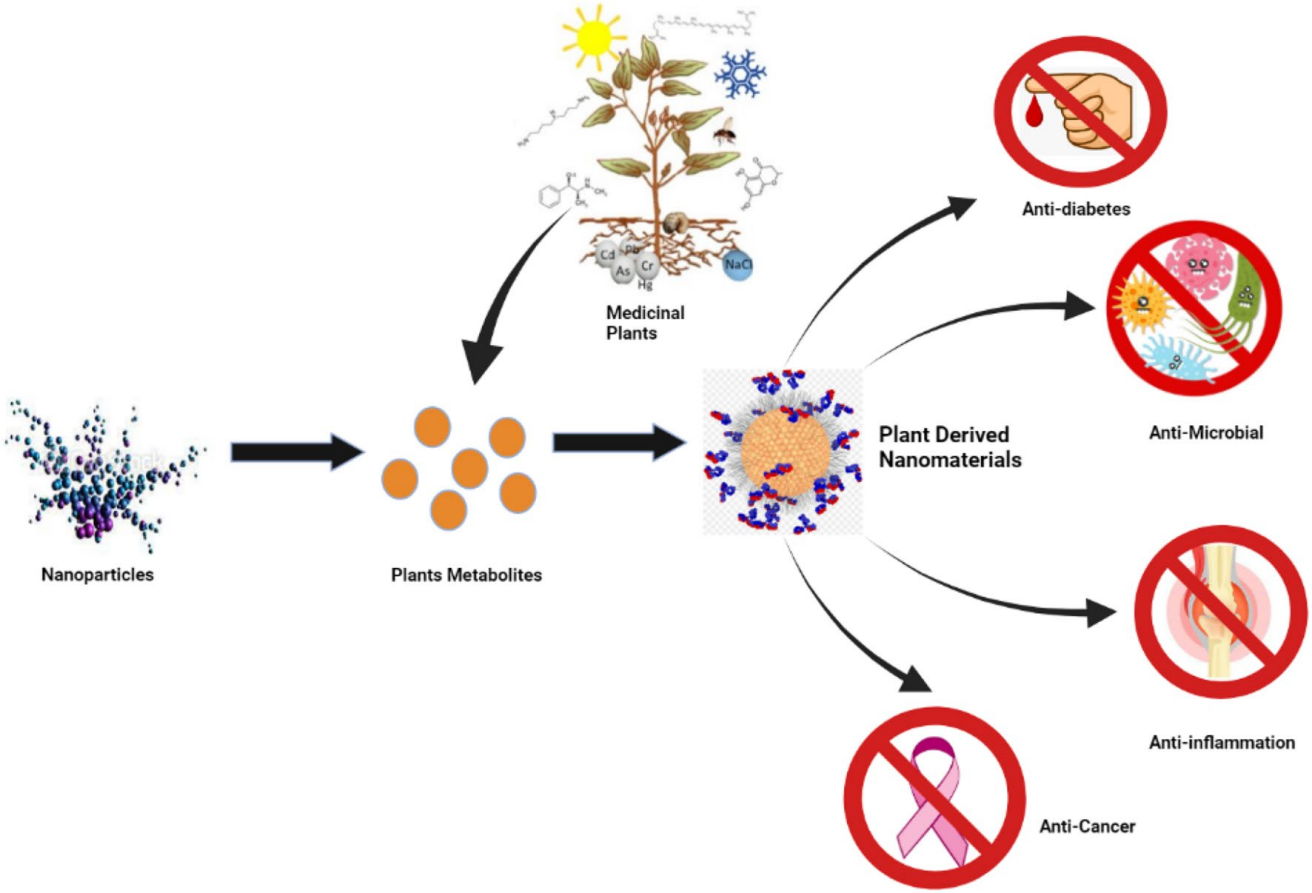
Biological activities/pharmacological potentials of plant-derived nanomaterials against pathogenic microbes and metabolic diseases

The antitumor activities of plant-derived nanomaterial could be attributed to their high surface area-to-volume ratio, which permits the presence of an atom on its surface and increased contact with the environment (Fig. 4). The adverse effects of the plant-derived gold nanoparticles were reduced, and the damage to normal cells was limited. This demonstrates nanoparticle aggregation and size-dependent cytotoxic effect against various cancerous cells, which also depends on the nanoparticle dose. Positive charges are present in gold nanoparticles, while negatively charged elements such as lipids are present in cancer cells, and these negative charges are responsible for both uptake and internalization (Patil and Kim 2016). A recent study shows that plant-derived silver nanoparticles had less genotoxic and cytotoxic effects than chemically synthesized silver nanoparticles (Patra et al. 2014). Recently, silver nanoparticles synthesized using *Nepeta deflersiana* plant extract showed anticancer activities against human cervical cancer cells, inducing concentration-dependent cytotoxicity in human cervical cancer cells as well as decreased glutathione levels (Al-Sheddi et al. 2018). Another independent study showed that plant-derived silver nanoparticles using leaf extract of *Cynara scolymus* employing photodynamic therapy showed anticancer activity via mitochondrial apoptosis in MCF7 cells. It was postulated that photodynamic therapy and plant-derived silver nanoparticle-induced intrinsic apoptotic pathways through the upregulation of Bax, a pro-apoptotic protein, and downregulation of antiapoptotic protein Bcl2 in breast cancer cells (Erdogan et al. 2019).

Numerous other studies report plant-derived nanoparticles' anticancerous activities (Abdel-Mohsen et al. 2014). *Morinda citrifolia root-*derived silver nanoparticles caused an antitumor effect against Hela cells, causing complete death of the cells at 100 μg of the silver nanoparticles (Suman et al. 2013). The anticancer activity of *Actinidia deliciosa*-derived silver nanoparticles against HCT-116 cells showed 78% viability (at 350 μg/mL) as well as cytotoxicity in a dose-dependent manner (Naraginti and Li 2017). The anticancer activity of plant-derived silver nanoparticles against the A549 cancer cell line showed an IC_50_ of 30 μg/mL (Lakshmanan et al. 2018). *Citrullus colocynthis-*derived silver nanoparticles caused a significant inhibition of cancerous cell proliferation in Hep 2 cell lines inhibiting cell viability to 50% at 500 nM (Satyavani et al. 2011). *Nigella sativa* leaves-derived silver nanoparticles were reported to reduce the viability of cancer cells in mouse bone marrow (Amooaghaie et al. 2015). *Melia dubia* leaf extract-derived silver nanoparticles showed a significant effect against the human breast cancer line with an IC50 of 31.2 μg/ml (Kathiravan et al. 2014). *Jurinea dolomiaea root and* leaf-derived nanoparticles showed anticancer activity against MCF-7 cell and HeLa cell lines (Ahmed et al. 2019). According to a study, AuNPs have been shown to have anticancerous activity against various cell lines, including the Hela, K-562, Vero, MDA-MB, and A-549 cell lines (Bhat et al. 2013). The primary mechanism by which the ZnO nanoparticles cause some cytotoxicity in cancer cells is by causing an increase in the release of dissolved zinc ions within the cell, which causes an increase in ROS generation and apoptotic cell death (Guo et al. 2013). A study used manufactured ZnO nanoparticles for a chemical precipitation method-based anticancer evaluation (Arakha et al. 2017). They showed that variously sized ZnO nanoparticles reduced fibrosarcoma HT1080 cell proliferation. TiO_2_ (titanium dioxide) nanoformulations’ potential method for preventing cell proliferation was proven in a study. According to research, TiO_2_ can stop a variety of DNA checkpoints during cell division, which can cause the cell cycle to break down (AshaRani et al. 2009). In addition to their research on tumor microenvironments, TiO_2_–NPs have also been linked to NAD salvage processes, neurodegenerative pathways, and redox homeostasis (Raja et al. 2018). Pure Au–TiO_2_–NPs, Zn–TiO_2_–NPs, and Ag–TiO_2_–NPs have been discovered to have the potential to develop into genotoxic pharmaceuticals when utilized as nanomedicines or chemotherapeutic agents to treat cancer, according to numerous recent research. To treat cancer tissues more successfully, radioactive probes can also be employed to penetrate deeply into them (Saeed et al. 2018; Iqbal et al. 2018). A study found that nanomedicine must go through circulation, aggregation, penetration, internalization, and release to reach tumor cells (Sun et al. 2017).

Plant-derived nanovesicles have several interesting properties that could be exploited in cancer treatment in four areas: selective apoptotic activation of tumor cells, inflammatory factor regulation, tumor microenvironment adjustment, and therapeutic drug delivery. TRAIL (tumor necrosis factor-related apoptosis-inducing ligand) is a critical target that promotes apoptosis in cancer cells while leaving healthy cells alone. Plant-derived nanovesicles have been shown to activate the TRAIL signaling pathway. A recent study shows that citrus lemon-derived nanovesicles generated from citrus lemon (size: 50–70 nm) caused significant inhibition of the proliferation of many types of solid and hematological cancer cells in vitro and suppressed the formation of chronic myeloid leukemia xenograft tumors in vivo (Raimondo et al. 2015). Recently, ginger-derived nanoparticles were shown to inhibit colitis-associated cancer via oral administration by significantly reducing the mRNA expression level of pro-inflammatory cytokines such as IL-1β and IL-6 and proliferation-mediated cyclin D1 (Zhang et al. 2016a). This finding shows that the ginger-derived nanoparticles inhibited colorectal carcinogenesis by lowering pro-inflammatory cytokine levels and regulating the metabolism of intestinal epithelial cells. Ginseng-derived nanoparticles significantly improved the ratio of M1/M2 of B16F10-allografted mice, effectively inhibiting melanoma growth via the alteration of macrophage polarization (Cao et al. 2019). Since most tumor types are associated with a lower M1/M2 ratio (Goswami et al. 2017). Lipids, bioengineered from grapefruit, were shown to facilitate the delivery of miRNA with miR-18a as a tumor suppressor to decrease liver metastasis by inducing M1 macrophages (Teng et al. 2016). *Astragalus tribuloides* Delile. root extract was used as a bioreduction and capping agent in a recent study to synthesize AgNPs employing a high-efficient, economical, green, and simple process (Sharifi-Rad et al. 2020). The study of the chemical processes used UV–Vis spectroscopy. Transmission electron microscopy (TEM), X-ray diffraction spectroscopy (XRD), and Fourier-transform infrared spectroscopy (FTIR) investigations were used to characterize the greenly produced AgNPs. The total phenolic and flavonoid contents, antibacterial, antioxidant, and anti-inflammatory properties of the *A. tribuloides* root extract and the greenly generated AgNPs were assessed (Sharifi-Rad et al. 2020). The outcomes showed that the AgNPs had an average size of 34.2 8.0 nm, spherical shape, and crystalline structure (Sharifi-Rad et al. 2020). AgNPs produced by green synthesis had lower total phenolic and flavonoid concentrations than *A. tribuloides* root extract. When compared with the antioxidant activity of the *A. tribuloides* root extract (47%), the produced AgNPs (64%) showed the proper antioxidant activity (Sharifi-Rad et al. 2020). The antibacterial test confirmed the increased bactericidal activity of the resultant AgNPs on Gram-positive and Gram-negative bacteria as compared to the *A. tribuloides* root extract. When compared with the *A. tribuloides* root extract, the greenly generated AgNPs had an odder anti-inflammatory impact (82% versus 69% at 500 g/mL). The *A. tribuloides* root extract was used to synthesize AgNPs, and these AgNPs generally exhibited good antibacterial, antioxidant, and anti-inflammatory properties, making them a prospective choice for several biological applications (Sharifi-Rad et al. 2020).

### Antisplasmodic activity

Vector control is very vital in an epidemic situation. Developing a cost-effective and environmentally friendly mosquito control strategy for nontarget organisms and the environment is critical. To control the vector mosquitoes, synthetic insecticides were employed, which might develop physiological resistance and harmful environmental impacts and be costly to prepare. To solve all the concerns, plant-based silver nanoparticles, which can rapidly be synthesized, are environmentally friendly as potential mosquito larvicidal agents [88]. Neem and Ashoka leaf extract-derived silver nanoparticles significantly inhibited the growth of *P. falciparum* in cell culture of human blood (Mishra et al. 2013). *Senna occidentalis* and *Ocimum basilicum* leaf extracts derived nanoparticles caused significant larvicidal and pupicidal activity against *Anopheles stephensi* and antiplasmodial activity against *Plasmodium falciparum* (Murugan et al. 2015)*. Nerium oleander* (Apocynaceae) leaf extract-derived silver nanoparticles showed a larvicidal effect against *Anopheles stephensi* due to easy nanoparticle penetration through the membrane. The larvicidal activity occurs due to the penetration of nanoparticles through a membrane (Roni et al. 2013). This proved to be a safe, easy, and environmentally friendly method of controlling mosquitoes serving as a novel strategy for vector control.

### Anti-inflammatory and antioxidant activities

Silver nanoparticles derived from Terminalia species are a rapid, green, and cost-effective way to make very stable silver nanoparticles. Flavonoids, phenolic acids, proteins, and polysaccharide compounds are abundant in Terminalia species and aid in the stability and production of silver nanoparticles. Due to capped phenolic and flavonoid molecules, these Terminalia species-derived nano-particles have antioxidant effects. It was also utilized to combat free radical damage and as an anti-inflammatory agent (Mohamed El-Rafie and Abdel-Aziz Hamed 2014). The presence of a considerable amount of phenolics, flavonoids, and trace elements like Selenium, Zinc, and Magnesium in *Uncaria rhyncophylla, Paeonia suffruticose, Tussilago farfara Sanguisorba officinalis, Spatholobus suberectus*, *Salvia miltiorrhiza,* and *Ligustrum lucidum* correlates with their antioxidant and anti-inflammatory properties (Ravipati et al. 2012). The extract of *Urtica pilulifera* showed antioxidant properties, inhibiting the oxidation of lipids via superoxide anion, metal chelation, reducing power, and H_2_O_2_ scavenging assays (Özen et al. 2010). The antioxidant capacity of *Terminalia chebula* extract-derived nanoparticle was evaluated, showing high reducing capacity and scavenging of hydrogen peroxide and nitric oxide-induced radicals dues to its high polyphenolic constituent (Saha and Verma 2018).

Plant-derived nanovesicles have been shown to play an essential role in intestinal immunological homeostasis by communicating with intestinal cells. Inflammatory bowel disease (IBD) is characterized by ulcerative colitis (UC). Owing to nonspecific pathogenic targeting and unavoidable damage to normal cells, traditional steroidal medicines and immunosuppressants have limited therapeutic results (Wang et al. 2014). As a result, nontoxic delivery systems which target colonic regions and have solid anti-inflammatory capabilities are critical for UC treatment. Grapefruit-derived nanovesicles exhibited an anti-inflammatory effect via the upregulation of heme oxygenase-1 and downregulation of TNF-α and IL-1β in the intestinal macrophages in the mice model (Wang et al. 2014).

Similarly, grapefruit-derived nanovesicles exhibited significant biodegradability, stability, and biocompatibility over a wide pH range. These characteristics allow grapefruit nanovesicles to act as a delicately engineered oral delivery system for anti-inflammatory medications like methotrexate (MTX), reducing cytotoxicity and improving therapeutic efficacy. Ginger-derived nanovesicles were used to develop a novel siRNA delivery system and applied for UC therapy (Zhang et al. 2017). Colon-derived nanovesicle were shown to regulate the colon immune system by regulating enzymatic pathways, such as activating AMP protein kinase to prevent dendritic cell activation in the intestine (Deng et al. 2017). Ginger rhizomes-derived nanovesicle suppressed downstream signaling inflammasome activation pathways like IL-18 secretion, interleukin (IL)-1β, pyroptotic cell death, and caspase1 autocleavage (Chen et al. 2019). The critical involvement of the lipid components of nanoparticles in blocking the assembly of NLRP3 inflammasomes is attributed to the molecular mechanism underlying the inhibitory effect mediated by ginger-rhizomes-derived nanoparticles. The results from a recent in vitro study to evaluate the anti-inflammatory properties of green synthesized silver NPs and the methanol extract of *Solanum khasianum* using the HRBC membrane stabilization assay technique shows that the green synthesized silver nanoparticle exhibited significant anti-inflammatory properties more than the methanolic leaf extract of the *S. khasianum* (Chirumamilla et al. 2022)*.*

**Fig. 5:**
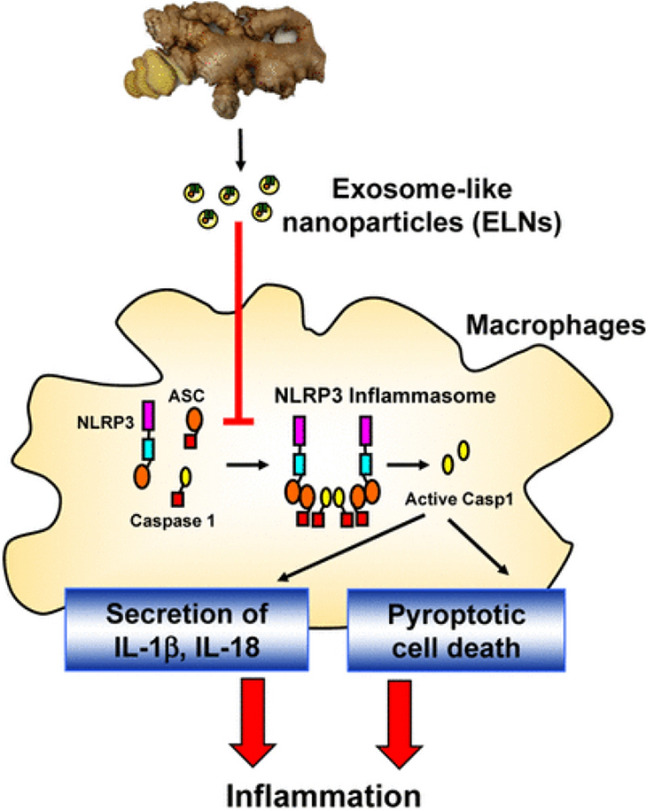
Diagram demonstrating the incubation of dietary ELN accompanied by inflammasome activation in Bone marrow-derived macrophages (Chen et al. 2019)

**Fig. 6:**
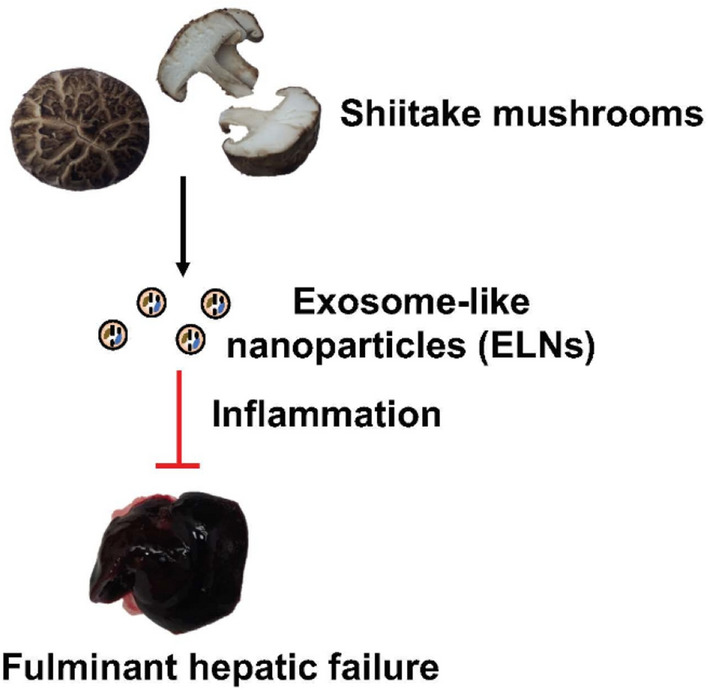
Schematic representation of significant inhibited the activation of the NLRP3 inflammasome by blocking the development of inflammasomes in primary macrophages by shiitake mushroom-derived ELNs

Exosome-like nanovesicles (NVs) from edible fruits and plants have modulated immune and inflammatory responses (Chen et al. 2019; Liu et al. 2020). A crucial regulator of innate immune responses, the nucleotide-binding domain and leucine-rich repeat family, pyrin domain-containing 3 (NLRP3) inflammasome is linked to the etiology of numerous disorders, including Alzheimer’s disease and type 2 diabetes. Although drugs that selectively inhibit the NLRP3 inflammasome have not been developed for patient therapy, targeting the NLRP3 inflammasome may hold promise in the fight against these complicated disorders. A recent study identified exosome-like nanoparticles (ELNs) in food that block NLRP3 inflammasome activity (Chen et al. 2019). Nine vegetables or fruits were chosen to extract ELNs, which were then tested for their ability to prevent the NLRP3 inflammasome from activating in primary macrophages. Although most of the evaluated ELNs had no effects, the ELNs from ginger rhizomes (G-ELNs) significantly reduced the activation of the NLRP3 inflammasome (Chen et al. 2019). The G-ELNs were readily ingested by macrophages and contained lipids, proteins, and RNAs. G-ELN therapy inhibited the release of IL-1 and IL-18 as well as pyroptotic cell death, which are downstream effects of inflammasome activation (Chen et al. 2019). G-ELNs prevented the NLRP3 inflammasome from being assembled, according to speck formation and oligomerization studies of the apoptotic speck protein with a caspase recruitment domain (ASC) (Chen et al. 2019). Instead of RNAs or proteins, the lipids in G-ELNs caused the inhibitory activity to be shown (Chen et al. 2019). Together, the information pointed to G-ELNs as potentially potent novel inhibitors of NLRP3 inflammasome construction and activation (Fig. 5). The development of G-ELN-based therapies to target the NLRP3 inflammasome in disease situations should be made more accessible by the special qualities of G-ELNs, such as biomolecule protection and tissue bioavailability (Chen et al. 2019). Fulminant hepatic failure (FHF) is a rare and fatal liver condition with a dismal prognosis (Panackel et al. 2015). In order to research prospective therapeutic treatments for FHF, lipopolysaccharide (LPS) and d-galactosamine (GalN) administration causes acute liver injury in mice that mimics several clinical characteristics of FHF in humans (Maes et al. 2016). Recently, it was demonstrated that inhibiting the NLR family, NLRP3 inflammasome might reduce the severity of liver injury in mice caused by GalN/LPS (Pourcet et al. 2018). A recent study aimed to identify dietary ELNs that could be therapeutically effective in reducing FHF by inhibiting the NLRP3 inflammasome (Liu et al. 2020). To extract ELNs, seven popular mushrooms were employed. It was discovered that these mushrooms had ELNs made up of proteins, lipids, and RNAs. Only the shiitake mushroom-derived ELNs (S-ELNs) significantly inhibited the activation of the NLRP3 inflammasome by blocking the development of inflammasomes in primary macrophages (Liu et al. 2020) (Fig. 6). IL-6 secretion, as well as the levels of the Il1b gene's protein and mRNA, were likewise reduced by S-ELNs (Liu et al. 2020). Surprisingly, pre-treatment with S-ELNs shielded mice from acute liver injury caused by GalN/LPS (Liu et al. 2020). S-ELNs, which have been discovered to be potent novel inhibitors of the NLRP3 inflammasome, are thus a promising group of therapeutics with the potential to treat FHF. Vesicle-like nanoparticles (H-VLNs), a new bioactive component, were recently discovered in honey (Chen et al. 2021). These H-VLNs are membrane-bound nanoparticles with proteins, lipids, and small-size RNAs as their main constituents. The presence of plasma transmembrane proteins and proteins linked with the plasma membrane suggests the probable vesicle-like character of these particles. The NLR family, NLRP3, and inflammasome, a critical inflammatory signaling platform in the innate immune system, are prevented from forming and becoming activated by H-VLNs (Chen et al. 2021). Mice with experimentally produced acute liver injury gain alleviation from inflammation and liver damage after receiving an intraperitoneal dose of H-VLNs. It was discovered that miR-4057 in H-VLNs inhibits the activation of the NLRP3 inflammasome (Chen et al. 2021). This research has revealed anti-inflammatory VLNs to be a novel bioactive component of honey (Chen et al. 2021).

### Antidiabetic activity

Recently, copper nanoparticles were reported to inhibit α-amylase and α-glucosidase, the key pharmacological targets of type 2 diabetes mellitus treatment. *Dioscorea bulbifera* tuber extract-derived nanoparticle inhibited α-amylase and α-glucosidase with a potent antioxidant capacity (Ghosh et al. 2015). The remarkable potency of these plant-derived copper nanoparticles for radical-scavenging and glycosidase inhibitory actions in vitro offered compelling scientific support for copper nanoparticles’ antidiabetic potential, which rationalizes their application in Type 2 diabetes mellitus therapy and management. *Lonicera japonica* leaf extract-derived nanoparticle caused a significant inhibition of intestinal a-glucosidase and pancreatic amylase, key digestive enzymes responsible for the breakdown of disaccharides and oligosaccharides into simple, digestible monosaccharides (Balan et al. 2016). Inhibition of these critical enzymes has proven to be particularly effective in treating noninsulin diabetes, as it limits the amount of glucose released into the bloodstream. *Tephrosia tinctoria*-derived nanoparticles showed antidiabetic activity through inhibiting enzymes involved in carbohydrate digestion and increased glucose absorption (Rajaram et al. 2015). This antidiabetic effect could be linked to its high phenol and flavonoid content. Nano-particles synthesized using *Solanum nigrum* were used in treating alloxan-induced diabetes mellitus in rats, resulting in improved dyslipidemic condition and reduced blood glucose levels (Sengottaiyan et al. 2015). The administration of ginger-derived nanoparticles (GDNP) in the drinking water of high-fat diet-fed mice for ≥ 1 year resulted in the restoration of balance in gut epithelium Foxa2 mediated signaling (HFD). Insulin resistance and obesity brought on by the HFD can be prevented by using GDNP therapy, which shields Foxa2 from being phosphorylated by Akt-1 (Ahmed et al. 2022; Kumar et al. 2022; Chopra et al. 2022). Although curcumin has poor solubility, absorption, and activity because of its physicochemical characteristics, it is one of conventional medicine's most investigated bioactive substances. Nanotechnology-based pharmaceutical formulations can increase curcumin's antidiabetic effects by overcoming its limited bioavailability. Numerous pharmacological routes that lessen the DMs defining hyperglycemia are responsible for nano curcumin's antidiabetic effects. Given these results, nanocurcumin may be considered a potential medication in the pharmacotherapeutic care of diabetes patients (Rahman et al. 2021; Quispe et al. 2022).

Another study found that the antidiabetic effects of ursolic acid contained in nanoparticles were significantly dose-dependent and improved glucose absorption by promoting the production of glucose transporter isoform 4 (GLUT4) (Castro et al. 2015; Singh et al. 2019a). The same study found that ursolic acid-loaded nanoparticles significantly reduced hyperlipidemia, which reduced insulin resistance. The study of betulin-loaded nanoparticles was prompted by betulin’s poor solubility and variable bioavailability, a well-known naturally occurring antidiabetic triterpene. When compared with the natural molecule, the betulin nanoparticles improved bioavailability and in vivo antidiabetic effectiveness (Zhao et al. 2014). Numerous studies have been conducted to establish the therapeutic benefits of glycyrrhizin, a triterpenoid saponin found in Glycyrrhiza plants (Seki et al. 2011). To increase its pharmacological characteristics, glycyrrhizin was put into nanoparticles. Glycyrrhizin loaded in nanoparticles showed significant antidiabetic and antihyperlipidemic properties in type 2 diabetic rats as compared to metformin, a traditional antidiabetic medication (Rani et al. 2017). In a different study, glycyrrhizin- and thymoquinone-loaded nanoparticles were combined to examine how they as compared to the individual formulation (Rani et al. 2018; Singh et al. 2019a). The in vivo antidiabetic efficacy of the combination formulations was significantly increased.

## Conclusion and prospects

This review highlighted the potential of medicinal plants for ethnopharmacological purposes, with a careful perspective on the link between antimicrobial activity, therapeutic ability, phytochemical, and traditional medicine applications. Medicinal plants and Ag-NP research are critical for various biological activities and medicinal uses. Plant-based silver nanoparticles can be used in multiple domains, including optics, electronics, and biology. Owing to their emerging potential, ag-NPs are also exploited as therapeutic platforms in biomedicine. Furthermore, hypertension and pulmonary arterial hypertension were treated with nanoparticles, nanoemulsions, and nanocapsules. The use of nanotechnology to distribute phytochemicals allowed traditional medications to connect with modern procedures and improve their antimicrobial and therapeutic efficacy.

However, one of the limitations of present investigations on the antibacterial processes of NPs is the lack of unified standards. Various bacterial strains, action timings, and NP properties have all been studied in other research, making comparisons of antibacterial activity challenging. Furthermore, no single method meets all the requirements for learning about NPs' antibacterial processes. Because different types of NPs have diverse bactericidal effects, a comprehensive examination of the putative antibacterial functions is frequently advocated. The antibacterial activity of NPs is frequently determined using sensitive bacterial strains.

The mechanisms through which NPs fight bacteria are currently unknown. For example, several studies link antibacterial activity to oxidative stress or ROS. However, the antibacterial agent for other NPs, such as MgO NPs, may not be related to bacterial metabolism regulation. As a result, future studies should focus on the antibacterial mechanisms of NPs.

Also, research into intracellular inhibitory pathways is still scarce. Few studies have looked at the effects of NPs on gene expression, protein synthesis, and metabolism in bacterial cells, although oxidative stress caused by NPs is well-known. Other drawbacks include the intricate structure of the bacterial cell membrane and a lack of research methodologies for in vitro studies. Besides, in vitro*,* models cannot completely mimic cellular interactions in the body since they cannot fully simulate the in vivo condition. As a result, estimating the antibacterial activity of NPs only through in vitro bacterial cell culture is impractical.

The concept of a controlled release of specific medications at certain places, as well as technology for assessing these events, drug effect in tissues/cells, and theoretical mathematical models of prediction, have yet to be perfected. Many nanomedicine studies focus on biomaterials and formulation investigations, which appear to be the early phases of biomedicine applications. Animal studies and transdisciplinary research, which demand a lot of time and research resources, will provide valuable data that might be used in drug therapeutic and diagnosis studies. Given the growing global trend toward more accurate medications and diagnoses, the future for a more intelligent and multi-centered approach to nanomedicine and nano-drug delivery technology appears bright.

Lastly, like their advantages, nanomedicines pose potential risks to humans and the ecosystem and require long-term research. As a result, a thorough assessment of the possible acute and chronic toxicity consequences of novel nanomaterials on humans and the environment is required.

## Data Availability

All data generated or analyzed during this study are included in this published article.
